# A Single-Tube HNB-Based Loop-Mediated Isothermal Amplification for the Robust Detection of the *Ostreid herpesvirus 1*

**DOI:** 10.3390/ijms21186605

**Published:** 2020-09-09

**Authors:** Maja A. Zaczek-Moczydłowska, Letitia Mohamed-Smith, Anna Toldrà, Chantelle Hooper, Mònica Campàs, M. Dolors Furones, Tim P. Bean, Katrina Campbell

**Affiliations:** 1 Institute for Global Food Security, School of Biological Sciences, Queen’s University Belfast, Belfast BT9 5DL, UK; L.Mohamed-Smith@qub.ac.uk; 2 IRTA, 43540 Sant Carles de la Ràpita, Spain; anna.toldra@irta.cat (A.T.); monica.campas@irta.cat (M.C.); dolors.furones@irta.cat (M.D.F.); 3 Centre for Environment, Fisheries and Aquaculture Science, Weymouth DT4 8UB, UK; chantelle.hooper@cefas.co.uk; 4 The Roslin Institute, The Royal (Dick) School of Veterinary Studies, University of Edinburgh, Midlothian EH25 9RG, UK; tim.bean@roslin.ed.ac.uk

**Keywords:** colorimetric, OsHV-1, LAMP, oyster, *C. gigas*, POCT, early warning detection

## Abstract

The *Ostreid herpesvirus 1* species affects shellfish, contributing significantly to high economic losses during production. To counteract the threat related to mortality, there is a need for the development of novel point-of-care testing (POCT) that can be implemented in aquaculture production to prevent disease outbreaks. In this study, a simple, rapid and specific colorimetric loop-mediated isothermal amplification (LAMP) assay has been developed for the detection of *Ostreid herpesvirus* *1* (OsHV-1) and its variants infecting *Crassostrea gigas* (*C. gigas*). The LAMP assay has been optimized to use hydroxynaphthol blue (HNB) for visual colorimetric distinction of positive and negative templates. The effect of an additional Tte UvrD helicase enzyme used in the reaction was also evaluated with an improved reaction time of 10 min. Additionally, this study provides a robust workflow for optimization of primers for uncultured viruses using designed target plasmid when DNA availability is limited.

## 1. Introduction

The *Herpesvirales* order comprises of three genetically distinct, linear double-stranded DNA (dsDNA) virus families including *Herpesviridae*, *Alloherpesviridae* and *Malacoherpesviridae*. These viruses share many characteristics including an icosahedral virion morphology [1], a relatively large genome ranging from 124,000–269,000 bp [2] and the capacity to establish latency [3]. Even subclinical levels of OsHV-1 in an asymptomatic oysters can be reactivated and can cause viral replication, suggesting that latency-like infection can exist in OsHV-1 infected oysters [4].

The *Malacoherpesviridae* family includes two genera of viruses, both of which infect mollusks: *Ostreavirus* and *Aurivirus* [5]. The *Ostreavirus* genus include OsHV-1 (and its µvar variants), which primarily infect *C. gigas* [6,7], and *Acute Viral Necrobiotic Virus*, which affects scallops such as *Chlamys farreri* [8]. The *Aurivirus* genera comprises of the *Haliotid herpesvirus 1* named Abalone herpesvirus (AbHV) [9].

OsHV-1 and variants have been identified globally as the cause of significant and prolonged disease outbreaks in cultured *C. gigas* since 2004 [10]. Once initial infection occurs on an aquaculture site, there are very few successful measures available to prevent the onset of disease and subsequent widespread mortality. As such, prevention and control through effective testing and biosecurity are paramount to reducing the impact of this pathogen [11]. Many countries, such as the UK, remain largely free of OsHV-1, and as such, measures to prevent import are rigorous within infected countries such as France [12,13]. Although mortality events are highly indicative, diagnosis of OsHV-1 as the cause of disease currently requires laboratory-based polymerase chain reaction (PCR) and sequencing to take place as per the World Organization for Animal Health (OIE) recommendations [13]. This process is accurate, sensitive and specific but also requires specialized laboratories and training and, in addition, may take several days to process, potentially delaying positive action. As such, rapid detection and diagnosis of OsHV-1 on site has the timely potential to allow early warning detection to prevent movement of infected animals and the subsequent spread of disease [14].

The current benchmark assay is based on PCR methods and is currently in use within the laboratory setting. The most commonly used conventional PCR primer set is C2/C6, designed on open reading frame (ORF) four, which covers a highly variable part of the genome, including the microsatellite locus [15,16]. The more sensitive real-time qPCR assay which targets the C region with C9/C10 primers can detect down to four copies of OsHV-1 per µL [17,18]. However, other amplification methods such as nucleic acid sequence-based amplification, helicase-dependent isothermal amplification (HDA), rolling circle amplification, multiple displacement amplification, recombinase polymerase amplification and LAMP are promising approaches. These isothermal methods are particularly applicable for the development of fast screening measures applicable in field or on-site conditions [19]. As well as high specificity and sensitivity, the above methods have rapid reaction times (approximately from 30–60 min) and relatively simple equipment requirements; amplification of DNA/RNA can be performed in thermo blocks or by using a water bath [19] and can optimized to relatively low temperatures [14].

In particular, LAMP assays have grown in popularity in the last decade and already have been employed for the detection of bacteria, viruses and fungi in healthcare, agriculture and veterinary sectors [20]. LAMP products can be visualized by the addition of SYBR™ Green, Picogreen, acridine orange, propidium iodide or calcein using methods such as visual assessment, gel electrophoresis, turbidity, fluorescence and absorbance measures [21,22]. The indicator HNB has been reported as a highly effective nontoxic alternative for visual assessment [20].

Thus far, two isothermal amplification primer sets have been developed to detect OsHV-1 from *C. gigas* [23] or *Scapharca subcrenata* [14]; however, both are specific only for OsHV-1 and require downstream visualization or the addition of dyes after amplification, which might lead to cross-contaminations. Here, we have developed a highly specific, robust and field applicable, one-tube HNB-based LAMP assay targeting OsHV-1 and its variants to achieve a multi-strain detection tool with prospective applications in aquaculture settings.

## 2. Results

### 2.1. OsHV-1 Specific LAMP Primer Design

Multiple alignments indicated that the region between 419–801 bp of ORF_4 was suitable for multi-strain detection, with the genetic differences of the ORF_4 gene among pairs of OsHV-1 variants ranging between 33.10–99.80% using pairwise comparison; therefore, the target was determined using ORF_4 sequences of OsHV-1 and µvar variants.

In silico evaluation of the three designed primer sets generated by the software included one pair of primers with stable DNA construct with no primer-dimer formation (Table 1 and Table S1).

The six designed primers indicated high specificity to detect OsHV-1 using basic local alignment search tool (BLAST) [24], with 100% identity and 100% query cover to OsHV-1 genomes available in the GenBank database (Table S1). For outer primers (forward-F3 and reverse-B3), no secondary structures were detected (Table S1). For inner primers (FIP and BIP), the possibility of occurrence of weak–moderate secondary structures was detected (Tables S1 and S2). Selected primers contained from 2 to 3 guanine or/and cytosine (GC clamp) bases in the last 5 bases (the 3′ end) (Table 1).

**Figure 1:**
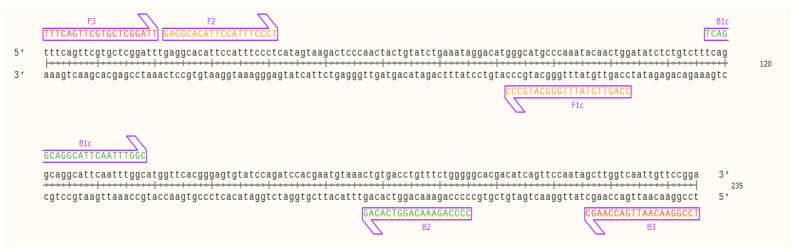
Nucleic acid sequence of the target OsHV-1 DNA fragment (235 bp) and designed LAMP primers binding sites; outer primers: F3 and B3 (red), inner primers: F2 and F1c (FIP) (orange), and B1c and B2 (BIP) (green) visualized using SnapGene Viewer® 5.0.4.

Evaluation of the primers by in silico hybridization indicated that the six primers annealed to distinct regions in 235 bp of the selected OsHV-1 target sequence: F1c (82–103 bp), F2 (22–41 bp), F3 (1–20 bp), B1c (117–138 bp), B2 (177–195 bp) and B3 (216–235 bp). Binding locations are indicated in Figure 1.

**Figure 2:**
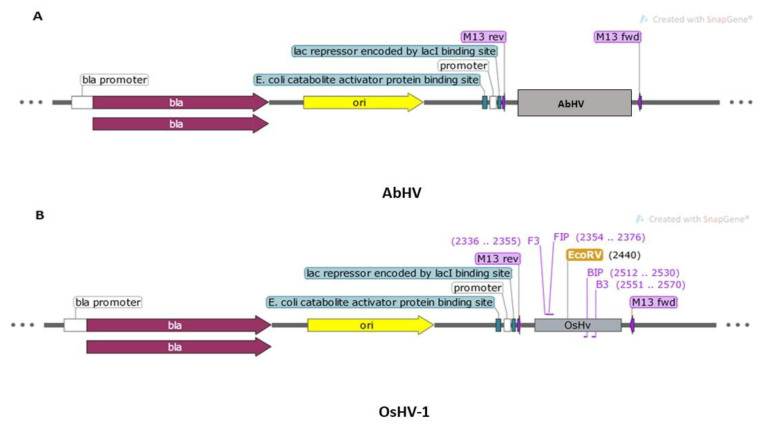
Maps showing the composition of two synthesized plasmids and primers binding to the target visualized using SnapGene 5.0.3.: (**A**) AbHV (3263 bp) and (**B**) OsHV-1 (3127 bp).

Prior to optimization of the primers in lab settings, in silico verification of the primer binding sites to the artificially constructed virus-target plasmids was performed. The analysis indicated no complementarity to the AbHV plasmid (Figure 2A) but showed complementary to the OsHV-1 plasmid in the region 2336–2570 bp (Figure 2B).

### 2.2. Bst DNA Polymerase LAMP Assay Optimization

**Figure 3:**
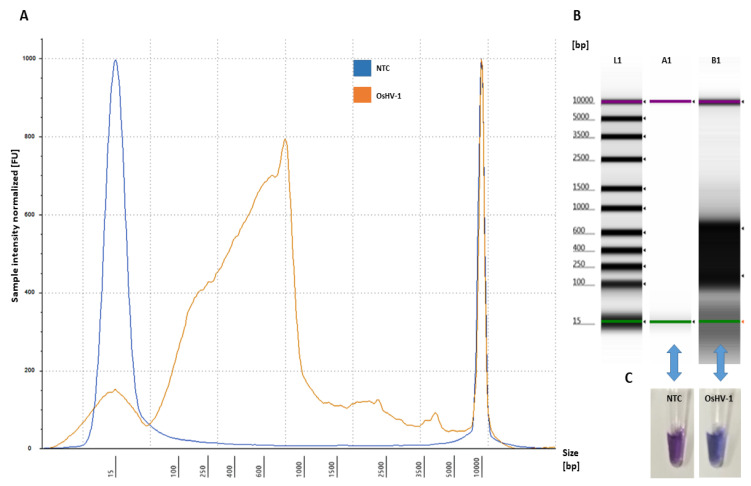
Amplification of OsHV-1 by LAMP assay: (**A**) electropherogram profile comparison of NTC (blue) and positive sample (OsHV-1 DNA-plasmid) (orange) analysed using Tape Station Analysis Software A.02.02 (Agilent Technologies, Cheshire, UK) with a progressive increase of the amplified LAMP product showing a size shift of 90–1000 bp; (**B**) gel image profile comparison: (L1)—ladder 15–5000 bp, (A1)—NTC and (B1)—positive samples showing a single high molecular band smear-like pattern (90 bp–1000 bp) of the amplified LAMP product visualized using Tape Station Analysis Software A.02.02 (Agilent Technologies, Cheshire, UK); and (**C**) NTC (violet) and positive samples (OsHV-1 DNA-plasmid) (blue) visualized using HNB.

Analysis of fluorescence intensity on electropherograms indicated that amplification of the OsHV-1 plasmid at three different DNA concentrations (1, 10 and 50 ng/µL) in the designed LAMP assay (Bst DNA polymerase; T = 65 °C, t = 60 min) was strongly dependent on a concentration of primers (Figure S1), with the optimal primer concentrations assessed for amplification at 1.6 mM for FIP/BIP primers and at 0.2 mM for F3/B3 primers (Figure 3). Experiments performed using two different concentrations deoxynucleotide (dNTP) solution mix (1.4 and 1.6 mM) indicated no difference with the increased yield of the LAMP product and successful amplification or for the colorimetric reaction using HNB (Figure S2). The concentration of MgSO_4_ and HNB in the performed reaction was crucial for visual assessment of the formed LAMP product, with 8 mM MgSO_4_ and 120 µM of HNB being the optimal concentrations (Figure S2).

The optimized assay for the designed LAMP primers using OsHV-1 DNA plasmid as a template showed that the amplified LAMP product yielded a maximum peak size formation between 90–1000 bp (Figure 3) and that no amplification peak was detected for the negative control (NTC) (Figure 3). Analysis of the obtained LAMP amplicon visualized on the electropherogram did not indicate low yield product, primer-dimers and artefact structures (Figure 3A). Visual assessment of the obtained LAMP product using HNB showed a blue product for positive and violet for NTC (Figure 3C).

After optimization of the reagent concentrations, the LAMP assay was also evaluated for determination of the optimal reaction temperature and time using HNB for a robust visual assessment. The colour change at the range of temperatures tested (63–65 °C) was consistent with the intensity of amplified OsHV-1 DNA, with 65 °C being the strongest (Figure S3). Afterwards, the LAMP assay performed at 65 °C was optimized by testing different reaction times. The most clearly positive results were obtained at 60 min, showing an intense blue colour, while the colour at 15–45 min was light to dark violet (Figure S3). Thus, the optimal reaction condition of the designed LAMP assay targeting the ORF_4 gene of OsHV-1 was 65 °C for 60 min (Figure S3).

### 2.3. Sensitivity and Applicability of LAMP Assay

**Figure 4:**
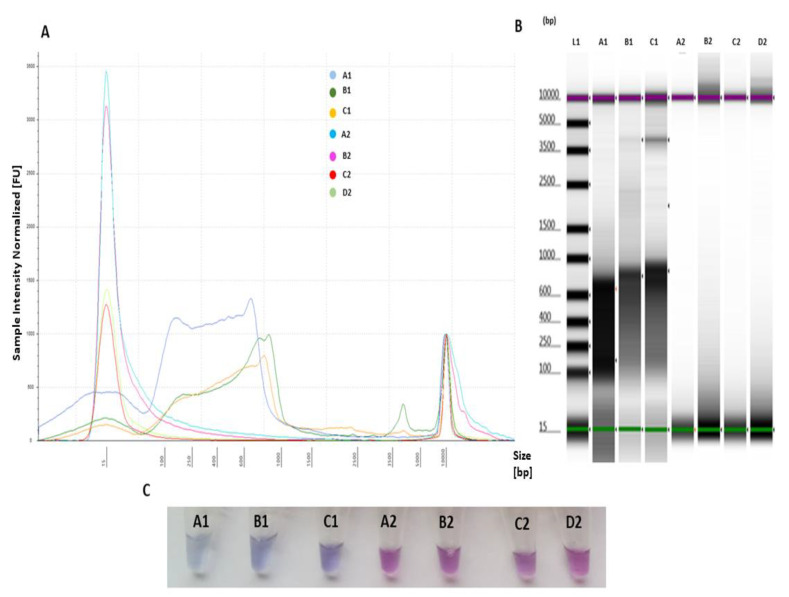
LAMP primer evaluation for specific target amplification and applicability to detect OsHV-1 present in the sample through comparison of positive and negative templates: A1—L_1 (10^3^ copies virus/reaction), B1—OsHV-1 plasmid (10^5^ copies virus/reaction), C1—L_3 (10^3^ copies virus/reaction), A2—NTC, B2—nuclease-free water (negative control), C2—UN_1 containing other DNA than the target (negative control) and D2—AbHV plasmid (negative control). (**A**) Electropherogram comparison profiles of positive and negative templates analysed using Tape Station Analysis Software A.02.02 (Agilent Technologies) for assessment of progressive increase of amplified LAMP product, with positive templates showing a size shift of 90–1000 bp; (**B**) gel image profile comparison of positive (A1, B1 and C1) and negative templates (A2, B2, C2 and D2) analysed using Tape Station Analysis Software A.02.02 (Agilent Technologies) for assessment of smear-like pattern bands; and (**C**) 6 colorimetric assessments of amplified LAMP products for positive samples (A1, B1 and C1) (light blue) and negative samples (A2, B2, C2 and D2) (violet) using HNB.

All positive samples (Table S3) were amplified by the LAMP assay, and no amplification was observed for negative samples (UN_1, AbHV and NTC). The profile comparison analysis of positive and negative samples visualized by electropherogram and gel indicated a size shift of 90–1000 bp for amplified positive and no amplification LAMP products for negative controls (Figure 4A,B). This was also confirmed by the LAMP assay performed for visualization of the samples using HNB, which showed three positive samples (light blue) (Figure 4C) and four negative results (violet) (Figure 4C).

**Figure 5:**
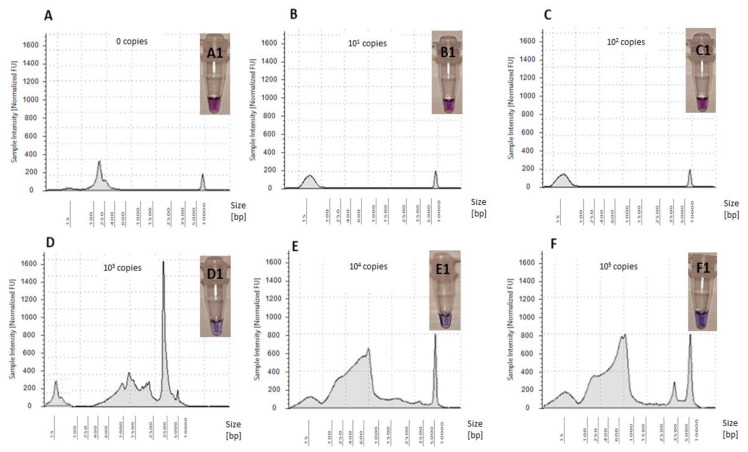
Electropherograms of NTC and five OsHV-1 plasmid standard amplifications in LAMP assay: (**A**) NTC—0 copies virus/reaction, (**B**) 10^1^ copies virus/reaction, (**C**) 10^2^ copies virus/reaction, (**D**) 10^3^ copies virus/reaction, (**E**) 10^4^ copies virus/reaction and (**F**) 10^5^ copies virus/reaction. Colorimetric assessment of amplified LAMP products using HNB: (**A1**) NTC—0 copies virus/reaction, (**B1**) 10^1^ copies virus/reaction, (**C1**) 10^2^ copies virus/reaction, (**D1**) 10^3^ copies virus/reaction, (**E1**) 10^4^ copies virus/reaction and (**F1**) 10^5^ copies virus/reaction.

The optimized LAMP assay was tested for five standards ranging from 10^1^–10^5^ copies/reaction using OsHV-1 plasmid as a template (Figure 5). Whilst amplification of OsHV-1 plasmid in comparison to NTC was observed when using standards ranging from 10^3^–10^5^ copies/reaction, no amplification was observed for standards ranging from 10^1^–10^2^ copies/reaction (Figure 5). Therefore, sensitivity of the assay was confirmed to be 10^3^ copies virus/reaction using Tape Station (Agilent Technologies) and visualized using the HNB assay (Figure 5).

### 2.4. Tte UvrD Helicase LAMP Assay

**Figure 6:**
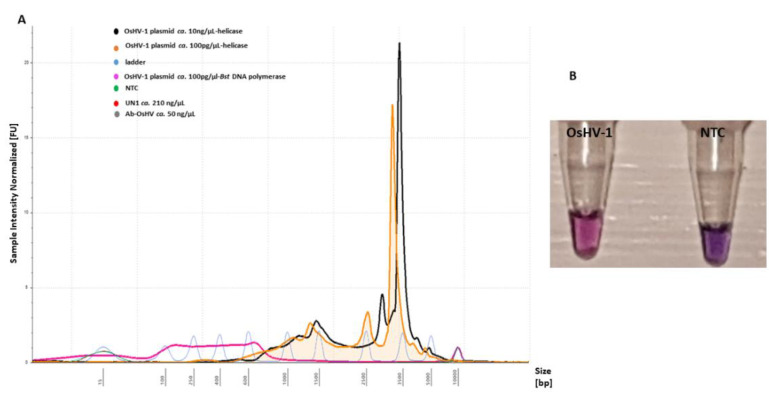
LAMP primer evaluation for amplification of the OsHV-1 plasmid (T = 65 °C, t = 10 min) and sensitivity with the addition of Tte UvrD helicase enzyme: (**A**) electropherogram comparison profiles of positive (OsHV-1 plasmid) and negative templates (NTC, UN_1 and AbHV) analysed using Tape Station Analysis Software A.02.02 (Agilent Technologies) for assessment of progressive increase of amplified LAMP product. Positive templates show a size shift of 350–5000 bp. (**B**) Colorimetric assessment of amplified LAMP products for positive (OsHV-1) (pink) and NTC (violet) templates using HNB.

Addition of a further enzyme (Tte UvrD helicase, ca. 20 ng/µL) to the LAMP assay resulted in colour change at t = 10 min, T = 65 °C with an intensive pink colour for positive and violet for a negative. In contrast, only the Bst DNA polymerase-based LAMP reaction did not show any colour changes after 15 min of amplification at T = 65 °C in comparison to the negative control (Figure S3A). An assessment of fluorescence intensity on the electropherogram indicated an unspecific progressive increase of the amplified LAMP product showing a size shift of 350–5000 bp with sharp high intensity peaks, which were not detected in the Bst DNA polymerase-based LAMP assay (Figure 6). The sensitivity of detection was evaluated to be 10^4^ copies virus/reaction, with no unspecific product formation with negative templates (Figure 6A).

## 3. Discussion

This study sought to design a specific and sensitive set of primers for the fast detection of several variants of OsHV-1 to prevent the spread in *C. gigas* populations by using it as an early warning tool in biosecurity protocols. In this study, the LAMP primers were designed based on a specific conserved OsHV-1 DNA region (ORF_4 gene encoding for DNA polymerase). Previously reported genome regions used for diagnostic of OsHV-1 include ORF_36_37_38, ORF_42_43, ORF_88, ORF_90, ORF_99, ORF_100 [25] and ORF_4, this latter showing more sequence divergence across the *Ostreavirus* than other genes [15]. Herein, interspecies variation of the ORF_4 sequence between OsHV-1 was taken into account when designing the specific LAMP primers (Figure S4) to target all variants of *Ostreavirus*. The six designed LAMP primers bound into a selected target when assessed for in silico hybridization (Figure 1) and in lab settings (Figure 3), thus in the presence of a single nucleotide polymorphisms, the primers should remain highly specific to the selected target [26].

Despite the designed primers being in the recommended range of GC content (40–60%) and 2–4 GC clamp to bind specifically to the target [27], in silico evaluation of two primers (FIP and BIP) showed moderate–weak secondary structures. Formation of secondary structures (i.e., hairpins) in LAMP long bases primers (40–45 bp) such as FIP and BIP frequently occurs, and it is not clear how these affect LAMP amplification [28]. However, there is the possibility that it can cause intermolecular/intramolecular interactions, which may decrease the yield or may affect primer amplification [29]. To counteract this risk, the LAMP assay optimized in this study contained betaine to increase specificity for detection only of the target; the assay evaluated here was shown to be highly specific to the target OsHV-1 template as evaluated in lab settings (Figure 4). Furthermore, in a previous study of LAMP tests to detect OsHV-1 T-bases, spacer sequences were used to improve LAMP efficiency by adding in loop formation with the FIP and BIP primers [23]. In this study, we investigated the effects of designed LAMP primers with (T4) linker spacer sequences for amplification efficiency associated with inserting T-bases and the designed primers that were specific to target OsHV-1 templates with no false positive results. Similar to another study, the (T4) linker spacer was the most efficient to improve specificity. In contrast, (T2) and (T6) linker spacers might decrease specificity of the designed primers by inhibition of loop formation during the self-priming process [30]. In this study, applicability of the LAMP assay was tested using samples obtained from OsHV-1-infected and uninfected pacific oyster spats and designed plasmids targeting ORF_4 gene. Analysis of fluorescence intensity using a Agilent 2200 Tape Station did not indicate amplification of negative templates, including a closely related genus (AbHV) or controls containing DNA other than OsHV-1 (UN_1; containing *C. gigas* DNA), as increase in yield LAMP product formation, suggesting specificity to the selected target. Moreover, no characteristic sharp peaks formed below the amplified LAMP product, indicating a lack of primer-dimer formation or cross-reaction with other targets. The sensitivity of the LAMP assay was assessed to be approximately 10^3^ copies virus per reaction of OsHV-1 plasmid and two field samples as evaluated by Agilent 2200 Tape Station and HNB visualization

In contrast to other dyes (i.e., berberine), the use of HNB dye has been shown recently as a promising nontoxic alternative for colorimetric visualization of LAMP amplicons, which can be assessed by several methods including visual assessment and gel electrophoresis [31]. Similar to several previous studies assessing HNB dye for detection of pathogens [32,33], herein, pre-addition of 120 µM HNB to the LAMP reaction solution did not inhibit amplification efficiency and a positive reaction was indicated by the easy discrimination of a colour change from violet (negative) to light blue (positive) and from pink (positive) to violet (negative) with the addition of helicase prior to amplification of the template. Another LAMP assay was previously developed targeting OsHV-1 [23]. Although visualization of the LAMP reaction using GeneFinder™ fluorescence dye was detectable by naked eyes, these colorimetric methods require additional steps including addition of dye to test for colour changes after reaction [23]. Therefore, this might decrease the utility of the LAMP assay in field settings and might lead to cross-contaminations. During LAMP amplification, cross-contamination might occur due to the possibility of *cis* and *trans* priming of primers, which can lead to nonspecific detection and false positive/negative results [34]. Thus, the pre-addition of HNB dye to the reaction tube prevents against potential cross-contamination that might have occurred. However, the pre-addition of HNB using the Agilent 2200 Tape Station was not possible. This analysis was performed in separate settings using Agilent 2200 Tape Station reagents, following the producer’s recommendations. As these contain another dye as an ingredient, that could cause interferences, experiments with HNB failed to detect the LAMP product. Agilent 2200 Tape Station is the most intensively used and recommended for quality control assessment of DNA/RNA and library preparation prior to whole genome sequencing [35], although it has not been explored so much for the evaluation of primers. In this study, it was evaluated for its efficiency to analyse LAMP products for the optimal concentration of reagents, primer-dimer formation and artefacts that are not easily detected or possibly visualised on gel by standard electrophoresis-based methods.

The HDA method was reported previously as an efficient POCT assay applicable in the field. HDA uses a DNA helicase i.e., *Escherichia coli* (*E. coli*)Tte UvrD helicase, to separate dsDNA with several benefits reported, such as unwind of dsDNA enzymatically, generation of single-stranded templates for primer hybridization, and subsequent extension or possibility to use the same optimized assays for detection of DNA or RNA [36]. In this study, the assay based on the HDA Tte UvrD helicase enzyme using six designed LAMP primers was tested for fast and specific detection of the OsHV-1 DNA target. Visual assessment of amplified LAMP products was possible after 10 min of amplification, with clear discrimination of positive and negative samples (Figure 6B). In contrast, the optimal amplification using only Bst DNA polymerase was reached after 60 min, although a shorter LAMP reaction time of 45 min has been reported previously [20]. This fast amplification using Tte UvrD helicase is in agreement with the producer (New England Biolabs) of the technical protocols ensuring a 10-min reaction [37]. However, in this study, LAMP Thermopol Master Mix was used instead of Warm Master Mix (New England Biolabs) as recommended, indicating efficient activity of the enzyme with Thermopol buffer and other ingredients composition. Interestingly, fluorescence intensity measured using Agilent 2200 Tape Station indicated an increase in the suppression of NTC and two other negative samples and a lower intensity for positive samples than Bst DNA polymerase-based LAMP assay (Figure 6A). As indicated previously, in the presence of Tte UvrD Helicase, the positive reaction maintains its rapid amplification time with a slight reduction in total fluorescence while the negative reaction is completely suppressed [37]. However, high intensity peaks might indicate that further optimization of primers or other reagents should be performed in this assay.

*C. gigas* is one of the main species produced in aquaculture, and its trade is of great economic importance and based both in hatchery produced spat and wild/naturalized animals. Until the OsHV-1 pandemic, *C. gigas* was supposed to be a very robust animal; however, the threat posed by OsHV-1 to this important production has challenged this sector. Thus, early warning tools, such as this robust one-tube LAMP assay, would be applicable in the field or under basic laboratory conditions allowing for fast decision-making regarding animal movements or relaying. In this study, applicability of the LAMP assay was tested only on three samples extracted from infected (L_1 and L_3) and uninfected (UN_1) oysters. However, such a tool could be extremely helpful both for producers and competent authorities in improving production performance and biosecurity in shellfish growing areas with further validation. Therefore, this LAMP assay should be further validated for usefulness in the field on a greater number of confirmed samples of OsHV-1 variants. Additionally, parallel double testing using qPCR and Agilent 2200 TapeStation would be useful to better understand the advantage offered by the addition of betaine in the LAMP assay and quantification possibilities of these two methods. This additional work could confirm the perspective use of the LAMP assay.

## 4. Materials and Methods

Invertebrate animals (except for cephalopods) are not included in the Directive 2010/63/EU of the European Parliament and of the Council of 22 September 2010 on the protection of animals used for scientific purposes. Thus, experimentation with oysters does not require approval by a research ethics committee.

### 4.1. Samples Preparation, DNA Extraction and qPCR

Three oyster homogenate lysates were used in this study for testing of the applicability of the designed LAMP assay (Table S3). The homogenates were prepared by mixing oysters (L_1: naturally OsHV-1-contaminated oysters during a mortality episode in 2018; L_3: naturally OsHV-1-contaminated oysters during a mortality episode in 2018; and UN_1: naïve spat oysters from IRTA hatchery) with UV-treated seawater and were homogenized using a stomacher for 1 min at maximum speed. After centrifugation (1000× *g*) for 10 min, the supernatant was collected. Lysates were filter-sterilized using a 10-mL syringe barrel fitted with a 0.22-µm filter Millex® GV filter unit (Merck, Darmstadt, Germany) and maintained in a 30-mL Sterilin® universal container (Thermo Fisher Scientific, Waltham, MA, USA) at −80 °C.

Prior to sequencing, a volume of 1 mL of each homogenate lysate was mixed with 2 mL of 16% polyethylene glycol (PEG) (Sigma Aldrich, St. Louis, MO, USA) (containing sterile 0.01 M phosphate-buffered saline, pH 7.2 and 79.89 g of PEG per 500 mL) and left at 4 °C overnight. After overnight incubation, propagated virion particles were concentrated and purified by ultra-centrifugation (113,000× *g*, 4 °C) for 6 h using the Beckman Sorral WX Ultra (Thermo Fisher Scientific, Waltham, MA, USA) ultracentrifuge. After centrifugation, the supernatant was discarded and tubes were left to dry for 20 min. The pellet containing virions was resuspended with 1 mL of nuclease-free water (Thermo Fisher Scientific, Waltham, MA, USA) and stored at −20 °C until use. Virus DNA was extracted using DNeasy® Blood and Tissue Kit (Qiagen, Manchester, UK), following the manufacturer’s instructions. Extracted DNA was stored at −20 °C prior to use. DNA extracts were tested for concentration and quality using a Nanodrop (Thermo Fisher Scientific, Waltham, MA, USA) or/and Agilent 2200 Tape Station and quantified with the method provided for the plasmid. For qPCR analysis, homogenate lysates were prepared with the method previously reported [38], were confirmed and were quantified using OsHVDPFor/OsHVDPRev primer set [39] using an ABI 7300 thermocycler (Thermo Fisher Scientific, Waltham, MA, USA) according to qPCR conditions reported previously [38].

### 4.2. DNA Sequencing Using MinION Device and Data Analysis

Prior sequencing of DNA extracts (L_1, L_3 and UN_1) (Table S3), Flongle Flow Cell (FLO-MIN 106) (Oxford Nanopore Technologies, Oxford, UK) was assessed for the number of nanopores available in the flow cell following the manufacturer’s instructions. For sequencing, a Rapid Sequencing Kit (SQK-RAD004) (Oxford Nanopore Technologies, Oxford, UK) containing Fragmentation Mix (FRA), Rapid Adapter (RAP), Sequencing Tether (SQT), Loading beads (LB) and Sequencing buffer (SQB) was used following the manufacturer’s instructions. Third-generation sequencing (TGS) was performed using a MinION device (Oxford Nanopore Technologies, Oxford, UK) with kit SQK-RAD004 (Oxford Nanopore Technologies, Oxford, UK) following the manufacturer’s instructions for sequencing. Data acquisition and real-time base-calling were performed using MinKNOW software (Oxford Nanopore Technologies, Oxford, UK), following the manufacturers recommended MinKNOW protocol. After sequencing, the FLO-MIN 106 was washed following the manufacturer’s instructions and stored at 4 °C prior to next analysis.

The obtained raw reads of the forward and reverse strands were paired into one single read list. The quality was enhanced by trimming off the low-quality reads using BBDuk tool, by correcting errors and by normalizing using BBnorm; chimeric and duplicate reads were removed using tools in Geneious Prime version 2020.2.2 (Biomatters Ltd., Auckland, New Zealand). Corrected sequences were assembled using de novo Geneious/Flye assembler and placed into scaffolds. Assembled genomes were compared with OsHV-1 genomes available in GenBank (National Centre of Biotechnology Information, Bethesda, MD, USA) using blastn tool in Geneious Prime version 2020.2.2 (Biomatters Ltd., Auckland, New Zealand) software.

### 4.3. Designing and Assessment of Synthetic DNA of OsHV-1 and AbHV

Synthetic DNA plasmids (5 µg) of two viruses (OsHV-1 with 3127 bp and AbHV with 3263 bp) were constructed based on a fragment of the ORF_4 gene in EcoRV cloning site and cloning vector Puc_57-BsaI-free. Lac promoter was used for the *E. coli* lac operon and synthesized (BioCat GmbH, Heidelberg, Germany). The number of OsHV-1 DNA copies in synthesized plasmid was calculated using following formula: number of copies = (amount × 6.022 × 10^23^)/(length × 1 × 10^9^ × 650) estimated from amplified concentration of the template using Agilent 2200 Tape Station and from the molecular weight of the input OsHV-1 target for the concentration tested. Plasmids were sequenced using the Sanger method using universal primers M13 and digested using restriction enzymes for product size verification. Obtained plasmid sequences were verified for specificity to OsHV-1 and AbHV through comparison with genomes available in GenBank using BLAST [24]. The binding sites of artificial plasmids to designed OsHV-1 primers were verified and visualized using SnapGene Viewer 5.0.3 (GSL Biotech LLC., Chicago, IL, USA) with hybridization parameters matching at least ten bases at the 3′ end of the primers and requiring melting temperatures at 40 °C. DNA plasmids were stored at −20 °C prior to use.

### 4.4. LAMP Primers Design and in Silico Specificity

Multiple alignment and pairwise comparison of 14 ORF_4 gene regions of OsHV-1 were performed using Geneious Prime 2020.2.2 (Biomatters Ltd., Auckland, New Zealand) for selection target region (Figure S4). Primers were designed based on the conserved region selected from sequence alignment of 14 DNA polymerase genes of OsHV-1 and variants (ORF 4, GenBank accessions AY509253, KY271630, KY242785, MG561751, KP412538, JQ959597, KM115665, KU518246, KM593669, KT429171, KT429177, KM886594, KY921758 and KX147758) (Figure S4) using the LAMP primer designing software PrimerExplorer V5 (Eiken) (https://primerexplorer.jp/e/v5_manual/index.html). The primer set was selected following guidelines for LAMP primers design and in silico optimization recommended by Lucigen® [27].

To improve loop formation efficiency between FIP and BIP primers, four thymine (T4) linker spacer sequence was employed between the F1c and F2, and B1c and B2 regions.

For all sets, the two outer primers (F3 and B3) were located outside the F2 and B2 regions. Designed primers were evaluated for secondary structures formation (hairpins, dimers, cross dimers, palindromes, repeats and runs), primers dimerization (primer-dimer), %GC and GC clamp using Oligo Evaluator™—Sequence Analysis and NetPrimer™ (PREMIER Biosoft International, San Francisco, CA, USA) (http://www.premierbiosoft.com/netprimer/). Each primer was assessed for specificity for detection) of OsHV-1 using BLAST [24].

### 4.5. Optimization of LAMP Conditions

Optimization of LAMP assay conditions was performed using synthetic OsHV-1 DNA plasmid in a total volume of 25 µL. The reagents were standardized in various concentrations (Table 2), which were previously reported as optimal reagents concentration for LAMP assays. Two different protocols based on ThermoPol Reaction Buffer Pack (New England BioLabs, Hitchin, UK) were assessed using two compatible enzymes: thermophilic Bst DNA polymerase large fragment (100% functional activity) (New England BioLabs, Hitchin, UK) and Tte UvrD helicase (100% functional activity) (New England BioLabs, Hitchin, UK). The assays were assessed based on HNB-visualized colour change and/or by using Agilent 2200 Tape Station (Agilent Technologies, Cheshire, UK) High Sensitivity D5000 DNA following manufacturer instructions. All samples were tested at least twice for confirmation.

#### 4.5.1. Bst DNA Polymerase LAMP Assay

For optimization of the LAMP reagents, the 25 µL amplification mixture contained 1× Thermopol Reaction Buffer Pack (B9004S) (New England BioLabs, Hitchin, UK), primers FIP/BIP (Thermo Fisher Scientific, Waltham, MA, USA), F3/B3 (Thermo Fisher Scientific, Waltham, MA, USA), dNTPs mix (Thermo Fisher Scientific, Waltham, MA, USA), 99% Ultra Pure betaine (Sigma Aldrich, St. Louis, MO, USA), MgSO_4_ (B10035) (New England BioLabs, Hitchin, UK), Bst DNA polymerase large fragment (M0275S) (New England BioLabs, Hitchin, UK), HNB dye 120 µM (Merck, Darmstadt, Germany) (Table 2) and 40 pg/µL of OsHV-1 DNA plasmid template (BioCat GmbH, Heidelberg, Germany). For analysis of LAMP product using Agilent 2200 Tape Station, the assay was performed in the same settings without HNB dye. A volume of 2 µL of LAMP product was added into 2 µL of High Sensitivity D5000 buffer (Agilent Technologies, Cheshire, UK) and visualized using Agilent 2200 Tape Station High Sensitivity D5000 DNA (Agilent Technologies, Cheshire, UK) tape to determine the optimal reaction conditions.

The LAMP assay was also optimized by testing different reaction temperatures (60, 63 and 65 °C) for 60 min in a thermo-cycler (Bio-Rad, Watford, UK) and then heated to 80 °C for 5 min to terminate the reaction. At the optimal temperature, the reaction time (15, 30, 45 and 60 min) was also evaluated and visualized using HNB dye.

All samples were tested at least twice for confirmation.

#### 4.5.2. Tte UvrD Helicase Lamp Assay

The total reaction volume of 25 μL contained 1.6 μM/µL of each FIP/BIP primer, 0.2 μM of each F3/B3 (Thermo Fisher Scientific, Waltham, MA, USA), 1.4 mM of dNTPs (Thermo Fisher Scientific, Waltham, MA, USA), 1 M of betaine (Sigma Aldrich, St. Louis, MO, USA), 8 mM of MgSO_4_ (New England BioLabs, Hitchin, UK), 1× ThermoPol reaction buffer (New England BioLabs, Hitchin, UK) (20 mM Tris–HCl, 10 mM KCl, 10 mM (NH4)_2_SO_4_ and 0.1% Triton X-100), 320 U/mL of Bst DNA polymerase large fragment (New England BioLabs, Hitchin, UK), Tte UvrD helicase 20 ng/mL (New England BioLabs, Hitchin, UK) HNB dye 120 µM and 2 μL of OsHV-1 DNA plasmid template (1 ng/μL). The reaction mixture was incubated 65 °C for 10 min in a thermo-cycler (Bio-Rad, Watford, UK) and then heated to 80 °C for 5 min to terminate the reaction. A volume of 2 µL of LAMP products was added into 2 µL of High Sensitivity D5000 buffer (Agilent Technologies, Cheshire, UK) and visualized using Agilent 2200 Tape Station High Sensitivity D5000 DNA tape (Agilent Technologies, Cheshire, UK) following manufacturers instruction. All samples were tested at least twice for confirmation.

### 4.6. Sensitivity and Applicability of LAMP Assay

After optimization of conditions for the LAMP assay using designed primers and OsHV-1 plasmid, the limit of detection was performed to determine the sensitivity of the LAMP assay. Synthetic genomic DNA plasmid of OsHV-1 was 10-fold serially diluted in nuclease-free water (Thermo Fisher Scientific, Waltham, MA, USA) in the range from 1 ng to 100 fg/µL and amplified as a template under the optimized LAMP assay conditions, following visualization using Agilent 2200 Tape Station and HNB dye.

To evaluate applicability of the LAMP assay, two positive samples (L_1 and L_3) and one negative sample (UN_1) containing DNA extracted from OsHV-1 contaminate and naïve *C. gigas* homogenates, respectively, were analysed. All samples were tested at least twice for confirmation.

## Figures and Tables

**Table 1:** Details of LAMP primers targeting OsHV-1 designed in this study.

Primer Name	Designed Primer/Sequence (5′–3′)	Length (bp)	GC (%)	GC Clamp
OsHV-LP-F	F3/TTTCAGTTCGTGCTCGGATT	20	45.0	2
OsHV-LP-B	B3/TCCGGAACAATTGACCAAGC	20	50.0	3
OsHV-LP-_FIP_^1^	F1c/CCAGTTGTATTTGGGCATGCCC	46	54.5	3
	F2/GAGGCACATTCCATTTCCCT			
OsHV-LP-_BIP_^2^	B1c/TCAGGCAGGCATTCAATTTGGC	45	48.9	3
	B2/CCCCAGAAACAGGTCACAG			

^1^ Forward inner primer (FIP) primer consisting of two inner primers: forward (F2) and reverse (F1c), synthesized with T4 linker spacer (F1c+TTTT+F2); ^2^ Backward inner primer (BIP) primer consisting of two inner primers: forward (B1c) and reverse (B2), synthesized with T4 linker spacer (B1c+TTTT+B2).

**Table 2:** Standardization of reagents used in LAMP assay.

No.	Reagent	Stock Concentration	Concentration of Tested Reagents/ 1 µL Reaction Volume		
1.	MgSO_4_ (New England BioLabs)	100 mM	4 mM	6 mM	8 mM *
2.	dNTP Mix (Thermo Fisher)	10 mM	n/a	**1.4 mM**	1.6 mM
3.	FIP/BIP Primers (Thermo Fisher)	40 µM	0.4 µM	0.8 µM	**1.6 µM**
4.	F3/B3 Primers (Thermo Fisher)	5 µM	0.05 µM	0.1 µM	**0.2 µM**
5.	HNB (Merck)	20 mM	120 µM	148 µM	400 µM

* Total optimized concentration of MgSO_4_ used in LAMP assay (2 mM Thermopol buffer + 6 mM MgSO_4_). The values (black bold) indicated optimal reagents concentrations selected for LAMP assay.

## Supplementary Materials

Supplementary materials can be found at https://www.mdpi.com/1422-0067/21/18/6605/s1. Figure S1: Electropherograms of primers concentration optimization for amplification of positive (OsHV-1) template in range of concentration, Figure S2: Colorimetric optimization of MgSO_4_ and dNTP concentration in LAMP assay, Figure S3: Colorimetric optimization of LAMP assay with the pre-addition of HNB-dye for optimal temperature and time of amplification, Figure S4: (A) Multiple alignment of 14 OsHV-1 ORF_4 gene gene fragments using MUSCLE tool in Geneious Prime 2020.1 (Biomatters). Red frame indicates aligned gene fragments (419–801 bp) used for construction of target sequences; (B) Pairwise comparison of 14 OsHV-1 ORF_4 gene fragments constructed using Geneious Prime 2020.1 (Biomatters). Black clusters indicate the highest percentage identities between sequences, Table S1: Additional details of designed LAMP primers in this study, Table S2: Secondary structures of BIP primer, Table S3: Details of samples used in this study.

## Abbreviations

LAMP	Loop-mediated isothermal amplification
HDA	Helicase-dependent isothermal amplification
HNB	Hydroxynaphtol blue
OsHV-1	*Ostreid herpesvirus 1*
AbHV	Abalone herpesvirus 1
PCR	Polymerase chain reaction
ORF	Open reading frame
