# Supplementary material for: A Single-Tube HNB-Based Loop-Mediated Isothermal Amplification for the Robust Detection of the Ostreid herpesvirus 1
Source: Int J Mol Sci. 2020 Sep 9;21(18):6605. doi: 10.3390/ijms21186605 (PMC7555478; doi:10.3390/ijms21186605)
Supplement: Supplementary file 1 [file ijms-21-06605-s001.zip › Supplementary materials-8.9.20/Table S1.docx]

**Table S1.** Additional details of designed LAMP primers in this study.

| **Primer name** | **Molecular Weight** | **Extinction Coefficient** | **µg/OD at 260 nm** | **Run Length (bp)** | **Primer Dimer** | **Secondary Structure** | **Id ^1^**  **(%)** |
| --- | --- | --- | --- | --- | --- | --- | --- |
| OsHV-F3 | 6105.1 | 180.6 | 33.8 | 3 | No | None | 100 |
| OsHV-B3 | 6095.1 | 195.4 | 31.2 | 2 | No | None | 100 |
| OsHV-FIP | 6717.4 | 200.3 | 33.5 | 3 | No | Moderate | 100 |
| OsHV-BIP | 13805.1 | 428.7 | 32.2 | 4 | No | Weak | 100 |

^1^ Percentage identity of primer sequence compared to OsHV-1 genomes available in GenBank using BLAST [24].
