# Supplementary material for: A Single-Tube HNB-Based Loop-Mediated Isothermal Amplification for the Robust Detection of the Ostreid herpesvirus 1
Source: Int J Mol Sci. 2020 Sep 9;21(18):6605. doi: 10.3390/ijms21186605 (PMC7555478; doi:10.3390/ijms21186605)
Supplement: Supplementary file 1 [file ijms-21-06605-s001.zip › Supplementary materials-8.9.20/Table S3.docx]

**Table S3.** Details of samples used in this study.

| **Sample Id.** | **Origin** | **OsHVDPFor & OsHVDPRev-*Ostreid herpesvirus 1* [39]** | **DNA sequencing** | **Species identified (Accession number)** |
| --- | --- | --- | --- | --- |
| L_1 | Alfacs Bay | + | + | *Ostreid herpesvirus 1*  (MT797824) |
| L_3 | Fangar Bay | + | + | *Ostreid herpesvirus 1*  (MT900589) |
| UN_1 | IRTA hatchery | - | - | *C. gigas* |

‘+’ tested positive with the method used; ‘-‘ tested negative with the method used.
