# Supplementary figures and images for: A Single-Tube HNB-Based Loop-Mediated Isothermal Amplification for the Robust Detection of the Ostreid herpesvirus 1
Source: Int J Mol Sci. 2020 Sep 9;21(18):6605. doi: 10.3390/ijms21186605 (PMC7555478; doi:10.3390/ijms21186605)

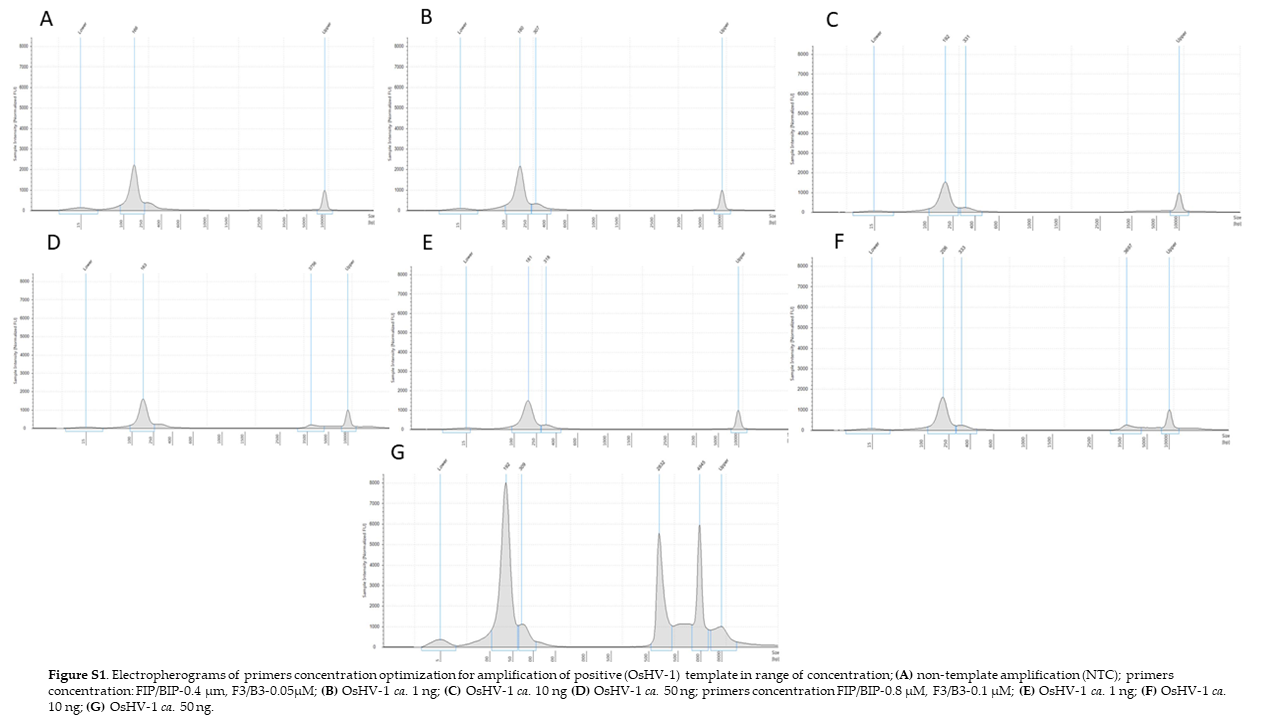

Supplement: Supplementary file 1 [file ijms-21-06605-s001.zip › Supplementary materials-8.9.20/Figure S1.tif]

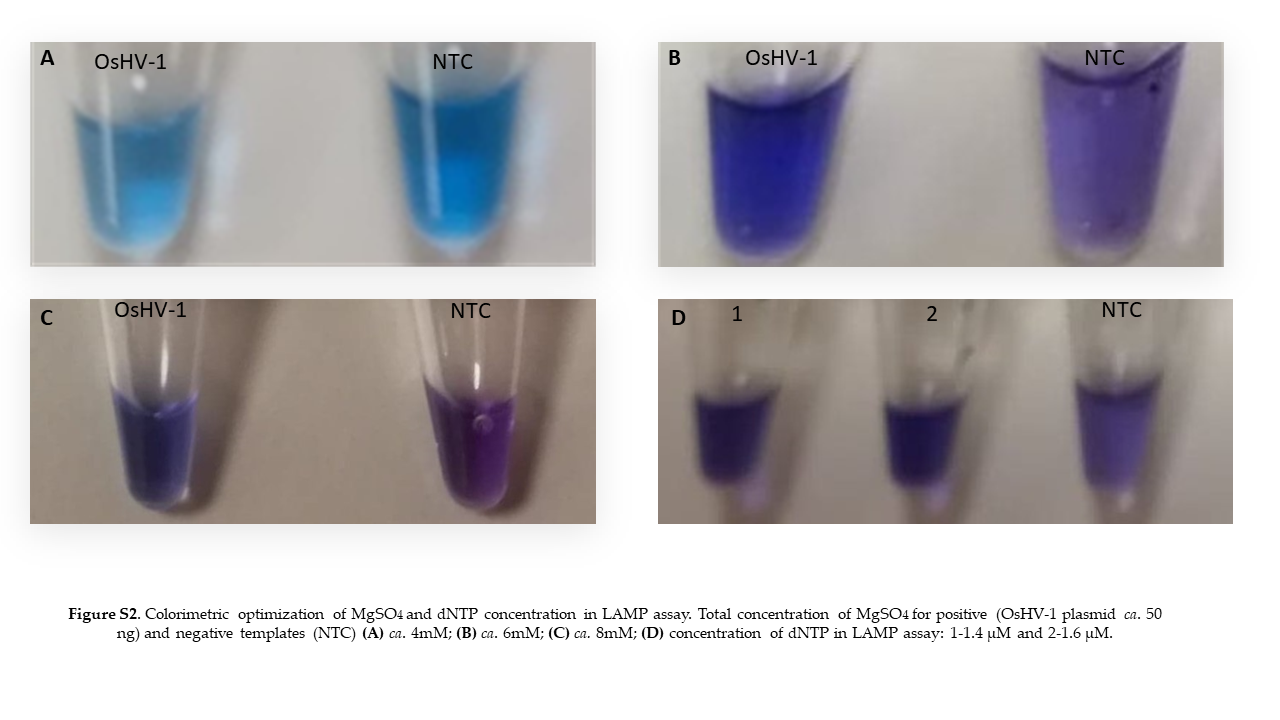

Supplement: Supplementary file 1 [file ijms-21-06605-s001.zip › Supplementary materials-8.9.20/Figure S2.tif]

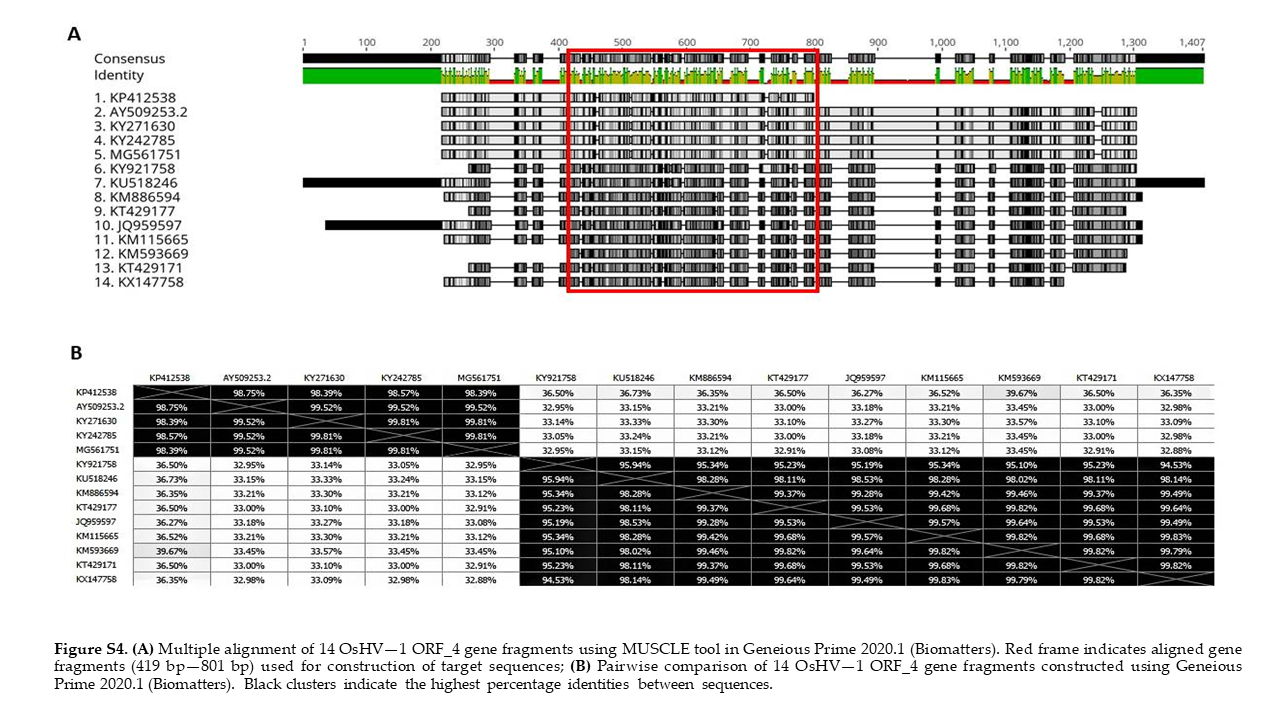

Supplement: Supplementary file 1 [file ijms-21-06605-s001.zip › Supplementary materials-8.9.20/Figure S4.tif]

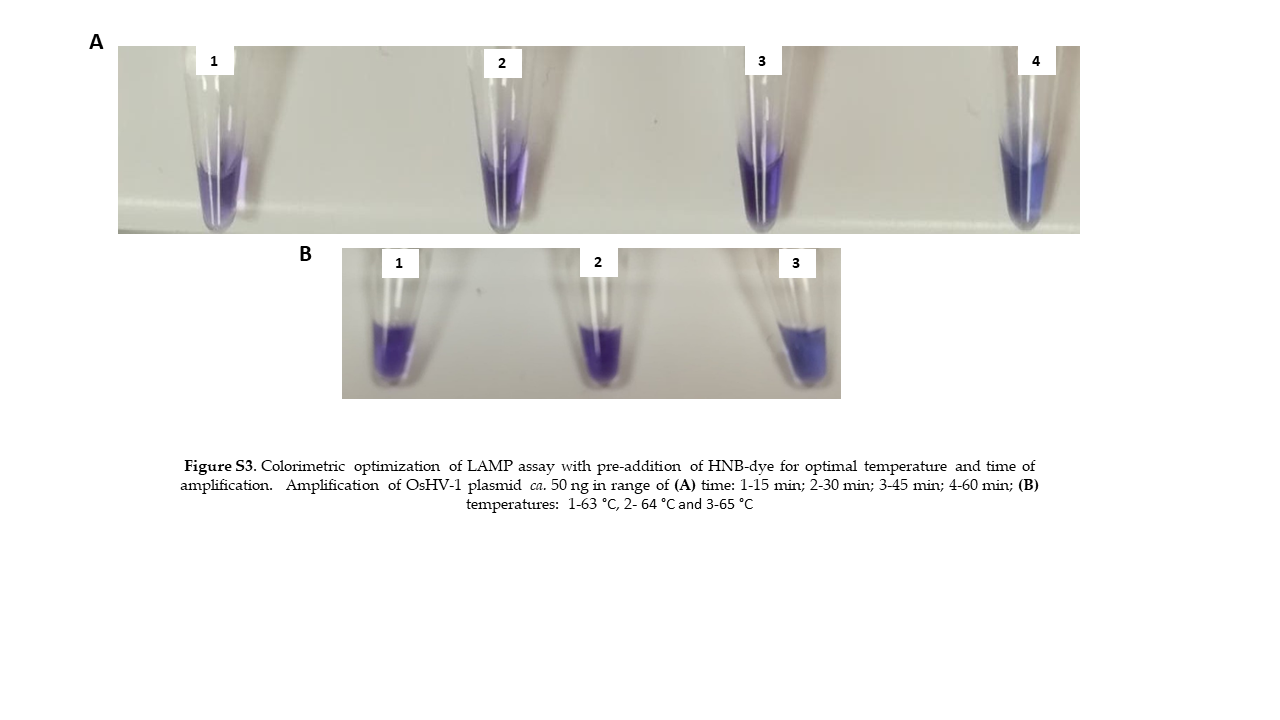

Supplement: Supplementary file 1 [file ijms-21-06605-s001.zip › Supplementary materials-8.9.20/FigureS3.tif]
